# Progress in enterovirus D68-neutralizing antibodies and vaccines

**DOI:** 10.1128/jvi.00123-26

**Published:** 2026-06-03

**Authors:** Liting Wang, Jun Shen

**Affiliations:** 1 Department of Infectious Disease, Children's Hospital of Fudan University, National Children's Medical Center 665527, Shanghai, China; 2 Pediatric Department, Children's Hospital of Fudan University at Qidong 145601, Nantong, China; Indiana University Bloomington, Bloomington, Indiana, USA

**Keywords:** enterovirus D68, neutralizing antibody, vaccine, antigenicity, acute flaccid myelitis

## Abstract

Enterovirus D68 (EV-D68) causes severe respiratory diseases and acute flaccid myelitis (AFM), for which no licensed vaccines or therapeutics are currently available. Neutralizing antibodies (nAbs) block infection via binding capsid epitopes. This review summarizes the EV-D68 antigenic structure, nAb mechanisms (receptor blockade and conformational disruption), therapeutic prospects of nAbs, and vaccine progress (inactivated, virus-like particle [VLP], and nucleic acid). Key challenges, like antigenic drift and blood-brain barrier (BBB) penetration, are also highlighted to guide countermeasure development.

## INTRODUCTION

EV-D68 is a non-enveloped, positive-sense, single-stranded RNA virus that belongs to the genus Enterovirus of the family *Picornaviridae*. First isolated from children with respiratory illness in California in 1962 (1), EV-D68 was historically considered a cause of mild respiratory infection. However, since 2004, EV-D68 has emerged as a pathogen of global concern, with large-scale outbreaks reported in North America, Europe, and Asia in 2014, 2016, and 2018 (2, 3). A defining feature of these recent epidemics is the strong association between EV-D68 infection and AFM, a devastating neurological disorder characterized by rapid-onset limb weakness and spinal motor neuron damage (4). The clinical severity of EV-D68-associated AFM, coupled with the absence of targeted therapeutics and vaccines, has elevated EV-D68 to a priority for biomedical research (5).

Neutralizing antibodies (nAbs) are the cornerstone of the adaptive immune response against EV-D68, mediating protection by targeting antigenic epitopes on the viral capsid to block infection (6). The effectiveness of nAbs is fundamentally governed by the structural architecture of the EV-D68 virion, which determines epitope accessibility and antigenic variation. Advances in structural biology techniques—notably cryo-electron microscopy (cryo-EM) and X-ray crystallography—combined with high-throughput single-cell antibody cloning, have substantially elucidated the molecular landscape of EV-D68 antigenicity and the modes of nAb recognition. Concurrently, seroepidemiological investigations have mapped the global distribution of EV-D68 immunity, highlighting distinct age-related and geographical patterns in antibody prevalence (7–11). Based on these insights, preclinical studies have established the therapeutic promise of nAbs, with multiple monoclonal antibodies (mAbs) demonstrating potent efficacy in protecting against EV-D68-mediated pathology *in vivo*.

## STRUCTURAL BASIS OF EV-D68 ANTIGENICITY

The EV-D68 capsid adopts the conserved icosahedral architecture typical of picornaviruses, with a diameter of approximately 27–30 nm. Composed of 60 identical protomers, each protomer comprises four structural proteins: VP1, VP2, VP3, and VP4 (12). VP1, VP2, and VP3 are surface-exposed and constitute the primary antigenic determinants recognized by nAbs, while VP4 is located internally, where it interacts with the viral genome and contributes to capsid stability (13). The spatial arrangement of these surface proteins, particularly the conformation of exposed loops and regions surrounding the icosahedral symmetry axes, defines the antigenic topography of EV-D68 and fundamentally determines the specificity of antibody recognition (14).

### Capsid architecture and key structural features

**Fig 1:**
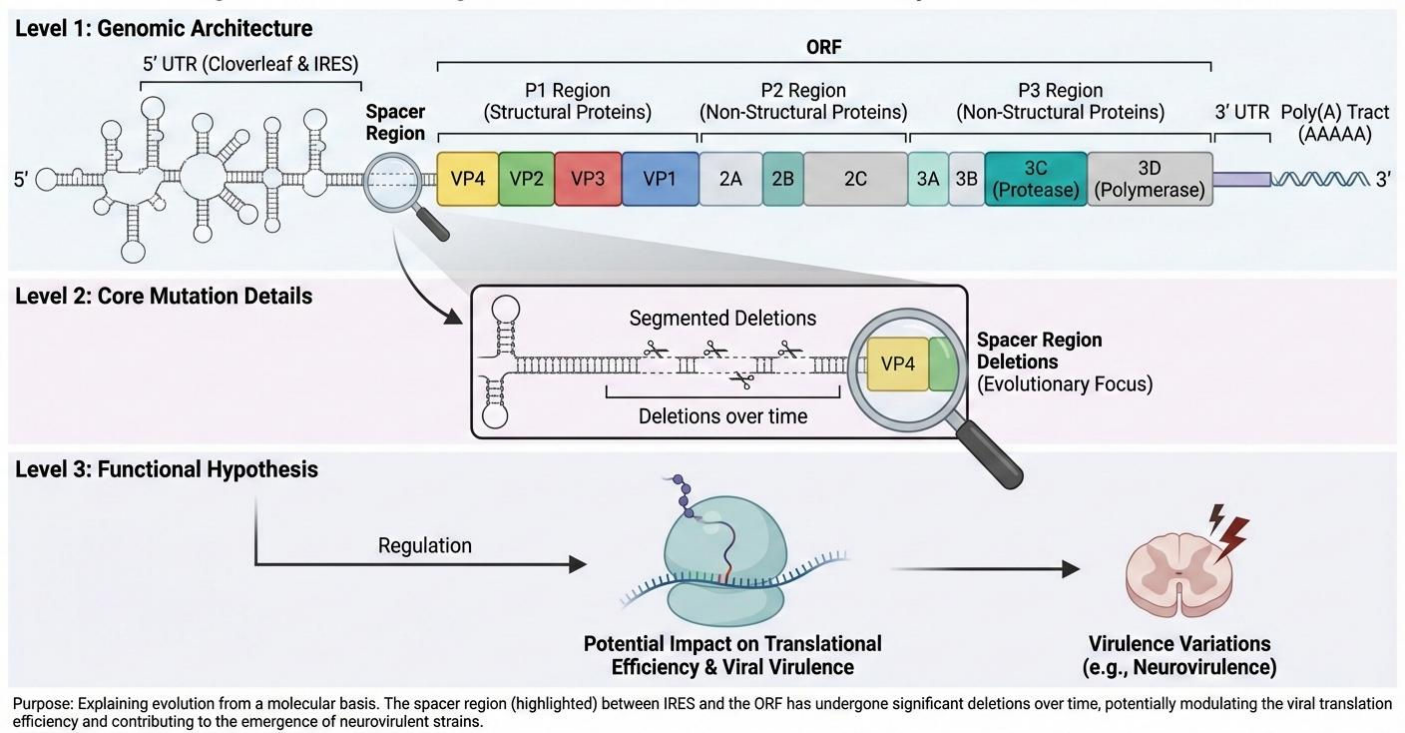
Genomic organization of EV-D68 and evolutionary deletions in the 5′ UTR.

The EV-D68 genome consists of a single open reading frame (ORF), encoding four structural proteins (VP1–VP4) and seven nonstructural proteins (2A–2C and 3A–3D), flanked by a long 5′ untranslated region (UTR) with a hairpin-loop secondary structure and a short 3′ UTR, followed by a poly (A) tract. Evolutionary analyses have revealed repeated deletions within a spacer region of the 5′ UTR, located between the end of the internal ribosome entry site (IRES) and the start of the polyprotein ORF. Although the functional consequences of these deletions remain unclear, they may modulate translational efficiency and could potentially influence viral virulence (Fig. 1) (15).

The EV-D68 capsid is organized around the three characteristic icosahedral symmetry axes (2-fold, 3-fold, and 5-fold), which give rise to distinct surface topographies that function as major epitope hotspots (16). Encircling the 5-fold axis is a pronounced, canyon-like depression—a structural hallmark conserved among picornaviruses—formed by loops from VP1, VP2, and VP3 (17). This canyon serves as the primary attachment site for host cellular receptors, and due to its relatively high sequence conservation across EV-D68 genotypes, it represents a key target for broadly nAbs. In contrast, the region surrounding the 3-fold axis forms a gently raised plateau (the threefold plateau) created by the convergence of VP2 and VP3 from neighboring protomers (18). This area displays greater sequence diversity among circulating EV-D68 strains, thereby contributing to antigenic variation and often strain-specific immune recognition (19).

**Fig 2:**
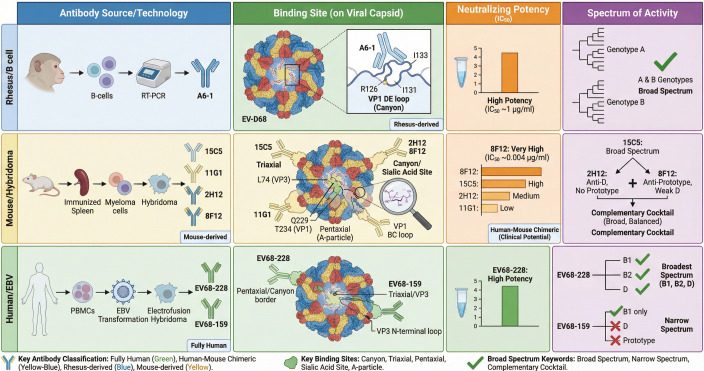
Comprehensive summary of EV-D68 nAbs.

VP1, the most immunodominant structural protein, contains several surface-exposed loops (BC, DE, and FG loops) that harbor both conserved and variable residues (Fig. 2) (20). The DE loop of VP1 contributes to the rim of the receptor-binding canyon, whereas the FG loop forms part of the 3-fold plateau. Although less immunodominant, VP2 and VP3 also play essential roles in antigenic site formation. The EF loop of VP2 and the BC loop of VP3, in particular, participate in the assembly of inter-protomeric (quaternary) epitopes that bridge adjacent capsid protomers (21). Such quaternary epitopes are especially significant for nAb recognition, as they are presented exclusively on the intact virion and not on isolated or denatured proteins, thereby conferring high specificity for infectious particles.

### Antigenic epitopes and neutralizing targets

Two principal classes of neutralizing epitopes have been defined on the EV-D68 capsid: those targeting the receptor-binding canyon and those directed against the 3-fold plateau (17). Antibodies that engage the canyon, such as EV68-228-N, typically bind conserved residues within this region, thereby sterically blocking viral attachment to host receptors (22). Cryo-EM structural analysis of the EV68-228-N Fab in complex with EV-D68 demonstrates that the antibody inserts its complementarity-determining regions deep into the canyon, establishing direct contacts with key residues in the VP1 DE loop and the VP2 EF loop—both of which are essential for receptor engagement (22). This mechanism of neutralization confers broad cross-genotypic activity, as the canyon region is highly conserved across EV-D68 genotypes (A–D).

In contrast, nAbs targeting the 3-fold plateau (e.g., 15C5, 11G1) typically engage variable, conformation-dependent epitopes, often formed by residues from VP2 and VP3 of adjoining protomers (14). The murine mAb 15C5, for instance, binds a quaternary epitope involving the VP2 HI loop and the VP3 BC loop. This binding induces conformational alterations in the capsid that interfere with the viral uncoating process (14). A major limitation of such plateau-targeting nAbs is their restricted breadth of neutralization, dictated by the inherent sequence variability of this region across EV-D68 strains. This is exemplified by the chimeric antibody 15C5-Chmra, which potently neutralizes prototype 2014 (genotype B) strains but shows no activity against 2018 isolates due to acquired mutations within the VP3 BC loop (21).

The structural principles governing epitope conservation and variability reveal a central paradox in EV-D68 immunology: the conserved canyon epitopes represent ideal targets for broad-spectrum nAbs; however, their recessed location may constrain natural immunogenicity. Conversely, the more exposed and immunogenic 3-fold plateau epitopes are susceptible to antigenic drift, limiting the breadth of antibodies they elicit (23). This fundamental trade-off between antigenic conservation and immunological accessibility critically shapes the host’s nAb repertoire and provides an essential framework for designing effective vaccines and antibody-based therapeutics.

### Global circulation and clade evolution

Based on phylogenetic analysis of the VP1 gene, EV-D68 has diversified into four major genetic clades (A–D) (24). Clade A represents the ancestral lineage, which includes the prototype Fermon strain but has been rarely detected in recent decades. Similarly, clade C, first identified in the United States around the year 2000, has shown limited contemporary circulation (25). In contrast, clade B emerged in the early 2000s and became the predominant lineage during the 2014 worldwide outbreak, with subclades B1, B2, and B3—particularly B3—circulating extensively across different regions (24). There is, however, continued co-circulation as recently as 2025 for the A2 lineage, which is often referred to as clade D (2, 26).

Clade evolution is driven primarily by amino acid substitutions in the surface-exposed capsid proteins VP1, VP2, and VP3. These mutations can alter epitope conformation and contribute to antigenic drift, potentially enabling immune evasion (27). For instance, the regional replacement of subclade B1 by subclade B3 has been linked to specific mutations within the VP1 DE and BC loops (27). Evolutionary rate estimation for VP1 (approximately 0.0039 ± 0.0001 substitutions per site per year) supports this ongoing diversification, which can diminish the neutralizing potency of antibodies elicited by earlier strains (19). Despite this antigenic variation in surface loops, the receptor-binding canyon remains structurally conserved, preserving a critical target for the development of cross-clade nAbs.

## EV-D68 nAb PREPARATION

The development of specific mAbs against EV-D68 offers a critical strategy for preventing and treating associated diseases. A range of potent EV-D68 nAbs have been obtained from diverse host sources through various preparation techniques (14, 23, 28, 29) (Table 1). These antibodies exhibit significant differences in antigen specificity, binding characteristics, neutralizing potency, and spectrum of activity while also demonstrating potential for functional complementarity (Fig. 2). This provides a rich pool of candidate resources and theoretical foundations for the development of antibody-based therapeutics.

**TABLE 1:** Summary of neutralizing antibody sources, binding sites, and core characteristics in EV-D68

Antibody name	Source	Preparation technology	Binding site/epitope	Target region	Core mechanism of action
EV68-228-N	Human (EV-D68-infected individuals)	PBMCs + EBV transformation + electrofusion hybridoma	VP1 DE loop, VP2 EF loop	Pentamer-canyon region	Blocking MFSD6 binding + capsid stabilization + immunomodulation
A6-1	Rhesus macaque (intranasally infected with EV-D68)	Flow sorting of VP1-specific memory B cells + RT PCR cloning	VP1 DE loop (near sialic acid-binding site)	Canyon region	Blocking sialic acid receptor binding
15C5	Mouse (immunized with EV-D68 clinical isolates/VLPs)	Hybridoma technology (splenocytes + myeloma cell fusion)	VP2 HI loop, VP3 BC loop (quaternary epitope)	3-fold plateau	Inducing capsid conversion to A-particles + blocking receptor binding
15C5-Chmra	Mouse-human chimera (modified from 15C5)	Humanization modification	VP2 HI loop, VP3 BC loop	3-fold plateau	Inducing capsid conformational change
8F12	Mouse (immunized with EV-D68 VLPs)	Hybridoma technology	Southern rim of canyon (overlapping with sialic acid receptor-binding site)	Canyon region	Blocking sialic acid binding + inducing conformational change
2H12	Mouse (immunized with EV-D68 VLPs)	Hybridoma technology	Canyon region (non-overlapping with 8F12)	Canyon region	Blocking receptor binding + perturbing capsid integrity
11G1	Mouse (immunized with EV-D68 clinical isolates)	Hybridoma technology	Pentamer region of A-particles (VP1 Q229, T234)	Pentamer region	Stabilizing A-particle conformation + preventing genome release
EV68-159	Human (EV-D68-infected individuals)	PBMCs + EBV transformation + electrofusion hybridoma	VP3 N-terminal loop + 3-fold region	3-fold plateau	Blocking sialic acid binding + stabilizing capsid to prevent uncoating

### Antibody sources and preparation technologies

From the perspective of antibody preparation sources and technical approaches, methods vary by host type. A6-1, the first EV-D68 mAb, was isolated from intranasally infected rhesus macaques: VP1-specific memory B cells were sorted via flow cytometry; antibody genes were recovered by RT-PCR and nested PCR and then recombinantly expressed and purified (28). Murine antibodies 15C5, 11G1, 2H12, and 8F12 were generated by hybridoma technology—immunizing BALB/c mice with clinical isolates or virus-like particles (VLPs), fusing splenocytes with myeloma cells, and screening; 2H12 and 8F12 were engineered into chimeric antibodies for clinical use (14, 23). Fully human EV68-228 and EV68-159 were obtained from EV-D68-infected individuals’ peripheral blood mononuclear cells (PBMCs): Epstein-Barr virus (EBV)-transformed memory B cells underwent electrofusion-based hybridoma generation, yielding naturally responsive nAbs with safety and low immunogenicity advantages (29). These strategies shape antibodies’ epitope specificity, neutralizing breadth, and therapeutic potential.

### Antigen binding characteristics and molecular mechanisms

The reported EV-D68-specific antibodies exhibit high binding specificity and distinct antigen-recognition profiles. All demonstrate strict specificity for EV-D68, with no detectable cross-reactivity against other enteroviruses such as EV-A71 or CV-A16 (14, 23, 28, 29). Although their epitopes are primarily located within key functional regions of the viral capsid, the precise localization and mode of recognition vary considerably.

A6-1 targets the conserved DE loop within the VP1-lined canyon, with residues R126, I131, and I133 identified as critical for binding (28). Its high affinity for the VP1 N-terminal region underlies its potent neutralization capacity (28). In contrast, 15C5 binds the 3-fold region of the capsid and recognizes multiple particle forms—mature virions, A-particles, and procapsids—with VP3 L74 serving as a key contact residue (14). 11G1 selectively binds A-particles at the 5-fold region, dependent on VP1 residues Q229 and T234 (14). EV68-159 also engages the 3-fold region, contacting both VP1 and VP3 (30). EV68-228 targets an epitope located between the fivefold axis and the canyon rim, corresponding to a classical neutralization immunogen site; a disulfide bond within its heavy-chain CDR3 is essential for maintaining the binding conformation (29). Both 2H12 and 8F12 competitively bind the southern rim of the canyon, with footprints overlapping the sialic-acid receptor binding site. Notably, 8F12 exhibits higher affinity due to additional contacts with the VP1 BC loop (23). These distinct binding characteristics directly determine the antibodies’ neutralization mechanisms—including receptor blockade and induction of conformational changes in the virion.

### Neutralizing potency and breadth

Neutralizing potency and breadth are critical parameters for evaluating the therapeutic potential of antibodies, and the reported EV-D68 nAbs show distinct stratification in both aspects.

In terms of potency, A6-1 exhibited 100% maximal percent inhibition (MPI) against clade A and B strains, with notably low IC₅₀ values of 1.57 μg/mL and 0.6 μg/mL, respectively, outperforming other early-described antibodies (28). The murine antibody 15C5 also showed high potency (IC₅₀ ~1.5 µg/mL), which was approximately 30-fold greater than that of the related antibody 11G1 (IC₅₀ ~39.7 µg/mL) (14). Similarly, 8F12 demonstrated exceptional affinity, neutralizing lineage B strains with an IC₅₀ as low as 0.004 μg/mL, substantially more potent than its counterpart 2H12 (23).

Regarding neutralization breadth, EV68-228 displayed outstanding cross-clade activity, effectively neutralizing strains from subclades B1, B2, and D with less than a 10-fold reduction in potency. In contrast, EV68-159 was largely restricted to subclade B1 strains, showing markedly reduced activity against clade D and prototype strains (28). A complementary neutralization profile was observed for 2H12 and 8F12:2H12 potently neutralized clade D strains but failed to neutralize prototype strains, while 8F12 showed the opposite pattern. Importantly, a 1:1 cocktail of these two antibodies successfully broadened the neutralization spectrum, achieving balanced and potent coverage against major circulating strains (23). These distinct profiles inform rational antibody selection for different applications: broadly, nAbs are suitable for general prophylaxis or treatment, while engineered combinations can overcome the limited breadth of individual antibodies.

The development and application of EV-D68 nAbs still have significant limitations. mAbs may lose clinical efficacy as the virus evolves, which is part of the impetus for considering polyclonal antibodies or even “cocktails” comprising mAb mixtures. Another consideration is that variant viruses may be selected under the nMAb pressure, although such variants are often attenuated rather than virulent; exceptions can occur.

## MECHANISMS OF nAb ACTION

**Fig 3:**
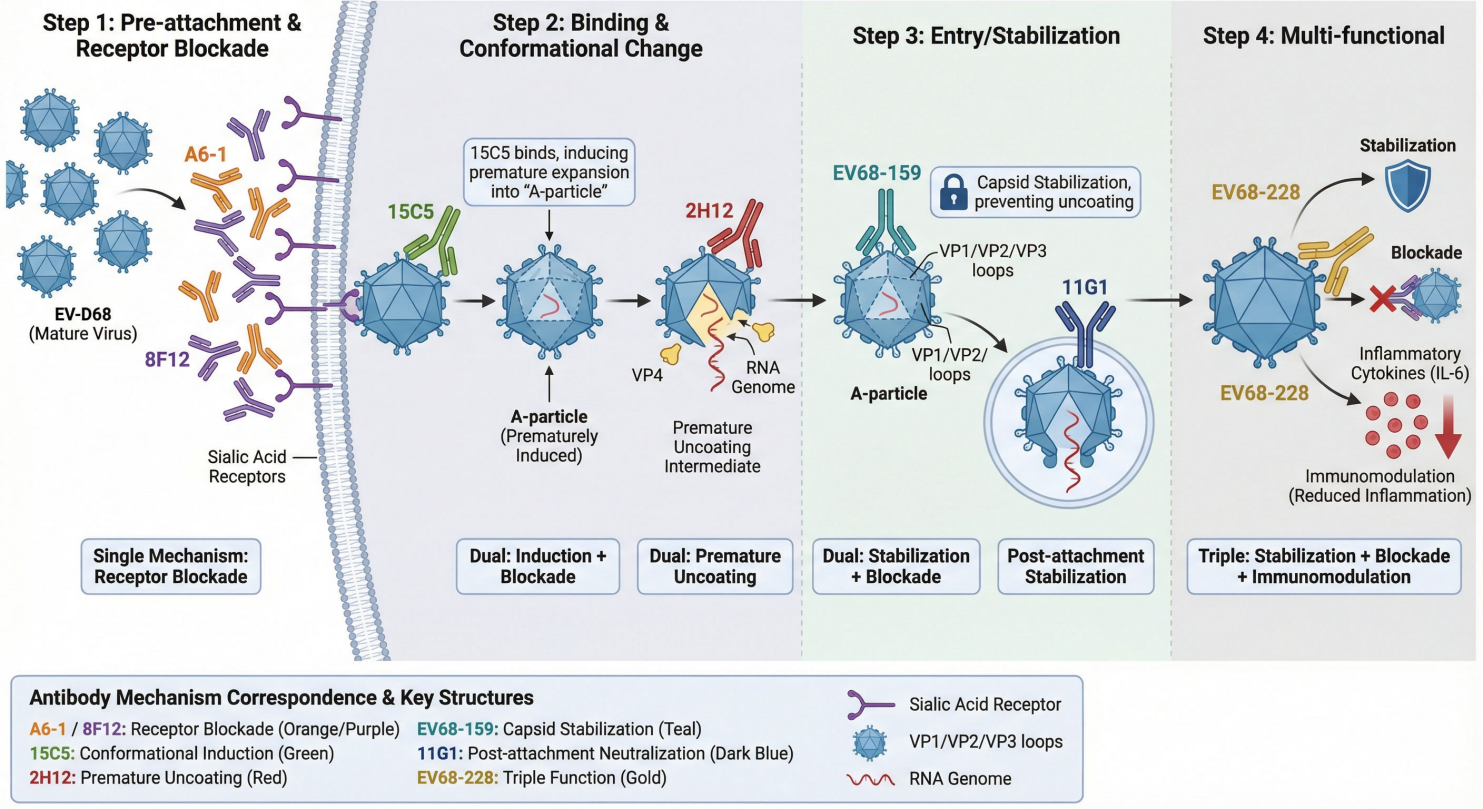
Hypothesized mechanism of action of EV-D68 nAbs.

EV-D68 nAbs inhibit viral infection by targeting key steps in the viral life cycle. This functional diversity reflects both the structural complexity of the viral capsid and the adaptable nature of the humoral immune response. The primary mechanisms of neutralization can be categorized into three core modes: blockade of cellular receptor attachment, induction of irreversible capsid conformational changes, and interference with viral uncoating. Individual antibodies may operate through a single mechanism or exhibit dual or multiple combined functions, resulting in synergistic antiviral effects (Fig. 3).

### Receptor blockade

Receptor blockade represents a direct and common mechanism of EV-D68 neutralization, whereby antibodies prevent the initial attachment of virions to host cell receptors via steric hindrance. The epitopes of such nAbs often overlap with or lie adjacent to the viral capsid regions that engage α2,6-linked sialic acid, the primary cellular receptor for EV-D68. Representative examples include A6-1 and 8F12. A6-1 binds near the sialic acid-binding site on the VP1 DE loop, inhibiting approximately 80% of viral attachment at 2.5 μg/mL and reducing both virion-cell association and receptor-particle colocalization (28). 8F12 recognizes an epitope that directly overlaps the receptor-binding region, dose-dependently suppressing EV-D68-mediated hemagglutination (23). Both antibodies are effective only when present prior to viral adsorption, and their neutralizing potency correlates directly with the efficiency of receptor occlusion. In addition, EV68-159 partially blocks receptor engagement through contacts with the VP1 C-terminus, thereby contributing to a dual mechanism of neutralization that also involves later steps in the viral lifecycle (29).

### Multiple mechanisms of action

Certain EV-D68 nAbs employ a dual mechanism of action, combining two distinct functional pathways to achieve enhanced neutralization. The antibody 15C5 binds the 3-fold region of mature virions. Its engagement induces a structural displacement of the VP2 HI and VP3 BC loops, triggering an irreversible expansion of the particle into an altered “A-particle” conformation with open pores at the 2-fold axes. This conformational change not only occludes the receptor-binding site through steric hindrance but also promotes premature genome exposure at 37°C, thereby neutralizing the virus through a combined blockade-and-uncoating mechanism. Similarly, 2H12 functions primarily through receptor blockade and also perturbs virion integrity (23). Its binding induces a cascade of conformational shifts, driving the particle into non-native states, including an expanded (S2) intermediate and an uncoating (S3) intermediate characterized by externalization of the VP1 N-terminus, loss of VP4, and reduced RNA density. This premature uncoating effect acts synergistically with receptor blockade to potently suppress infection. Another example is the human-derived antibody EV68-159, which employs an attachment inhibition plus uncoating blockade strategy (29). While directly interfering with sialic acid receptor engagement, it also stabilizes the capsid by binding the VP3 N-terminal loop, thereby preventing the structural rearrangements and VP1 N-terminal externalization required for successful uncoating (30). Notably, the VP1 C-terminal epitope recognized by EV68-159 shows high homology to epitopes targeted by antibodies found in the cerebrospinal fluid of AFM patients, offering important clues regarding viral neurotropism (29).

Some antibodies exhibit stage-specific or multi-mechanistic properties that broaden the functional scope of classical neutralization. The antibody 11G1 employs an adsorption-stage neutralization strategy: it specifically recognizes A-particles generated after receptor engagement rather than mature virions (14). By binding near the 5-fold axis and stabilizing a disordered loop structure in VP1, it prevents genome release from the capsid into the cytoplasm. Remarkably, 11G1 retains neutralizing activity even after the virus has attached to cells (IC₅₀ ≈ 39.0 μg/mL), demonstrating the feasibility of targeting post-attachment steps for therapeutic intervention (14).

The human antibody EV68-228 represents a case of triple-function integration. Its binding to the 5-fold region stabilizes the capsid to inhibit uncoating and genome ejection, while also sterically hindering receptor interaction. Beyond these direct antiviral effects, EV68-228 displays immunomodulatory activity: in infected mice, it significantly reduces pulmonary levels of pro-inflammatory cytokines such as IL-1α, IL-6, and MCP-1, thereby mitigating inflammatory tissue damage (29). This combination of viral neutralization and immune regulation overcomes the limitation of conventional nAbs that target only the virus, resulting in enhanced *in vivo* protective efficacy.

In summary, the mechanisms of EV-D68 neutralization demonstrate a progression from simple steric blockade to sophisticated multifunctional synergy. The diversity and complementarity of these mechanisms—reflected in their distinct stages of action, epitope specificities, and functional outcomes—not only advance our molecular understanding of virus–antibody interactions but also provide a robust conceptual framework and versatile strategic options for the rational design of high-efficacy nAb therapeutics.

## INTRACORPOREAL PROTECTIVE EFFECT

**Fig 4:**
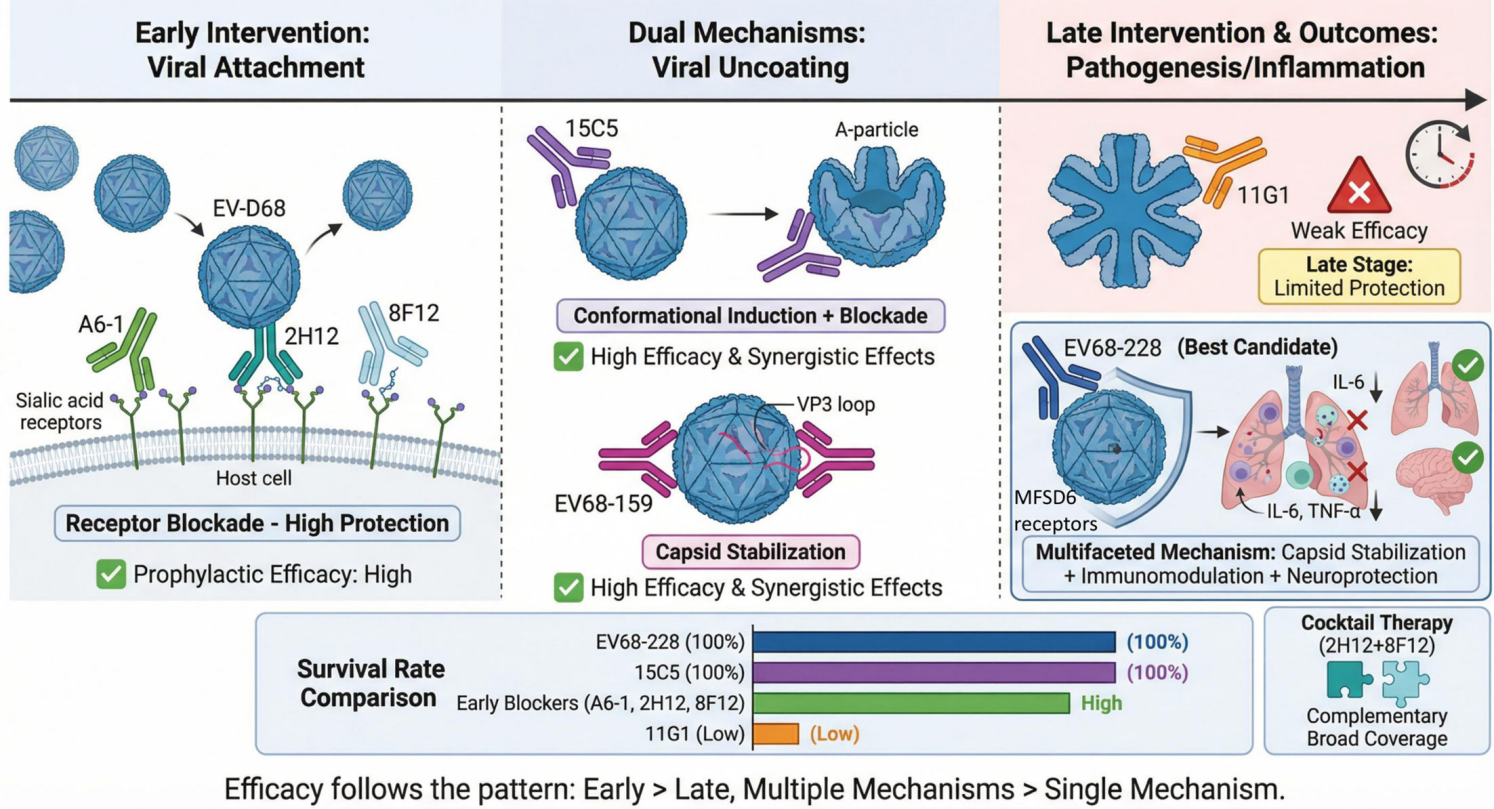
Intracorporeal protective effect of nAbs.

The *in vivo* protective efficacy of EV-D68 nAbs varies considerably and is governed by multiple factors, including their molecular mechanisms, specific stages of intervention, and epitope targets. Additional variables such as administration timing, dosage, and viral strain characteristics further modulate protective outcomes. Understanding these determinants provides a critical foundation for designing effective clinical application strategies (Fig. 4).

### Antiviral mechanisms

The protective efficacy of EV-D68 nAbs *in vivo* is closely linked to their antiviral mechanisms, with antibodies that intervene early in the viral life cycle—particularly at pre-attachment or mature virion stages—typically demonstrating robust protection. The receptor-blocking antibody A6-1 illustrates the preventive advantage of inhibiting viral attachment. Prophylactic administration of A6-1 in rhesus macaques and murine models completely suppresses viral replication in both lung and brain tissues, attenuates pathological damage, and provides effective protection against central nervous system (CNS) infection (28). Leveraging its dual mechanism of conformational induction plus receptor blockade, 15C5 achieves 100% survival in BALB/c mice immunized with EV-D68 clinical isolates or VLPs in murine models under both prophylactic and therapeutic regimens. By preventing attachment and irreversibly altering virion conformation, it exhibits superior *in vivo* neutralization compared to antibodies relying on a single mechanism (14). The human-derived antibody EV68-228 combines multiple functions to deliver comprehensive protection. Beyond capsid stabilization and receptor interference, its immunomodulatory capacity reduces pulmonary inflammation in neonatal Swiss Webster mice and murine models of EV-D68-induced AFM. It confers protection against CNS infection in both prevention and delayed-treatment settings, with measurable neurological improvement observed within 72 h post-treatment. Notably, EV68-228 shows no evidence of antibody-dependent enhancement (ADE) and a low propensity to select for escape mutants, underscoring its strong clinical translation potential (29).

### Therapeutic window

The therapeutic window critically governs the *in vivo* efficacy of antibody-based interventions. 11G1, which employs a post-attachment neutralization strategy, acts only after A-particle formation and cannot prevent initial viral binding (14). Consequently, even prophylactic administration merely delays disease progression without preventing lethal infection, underscoring the limited efficacy of late-stage intervention. The comparative study of 2H12 and 8F12 demonstrates that even when sharing the primary mechanism of receptor blockade, additional functional properties and strain-specific adaptations can significantly enhance *in vivo* protection (23). Both antibodies exhibit a broad therapeutic window, sustaining high survival rates even when administered post-symptom onset.

In summary, the *in vivo* protective efficacy of EV-D68 nAbs adheres to several guiding principles: multifunctional mechanisms outperform single-mechanism approaches; early intervention surpasses late-stage blockade; and broad-spectrum adaptability offers greater clinical utility than strain-restricted specificity. Antibodies capable of early blockade, multi-step inhibition, and immunomodulation—such as EV68-228 and 15C5—consistently demonstrate superior protective profiles. A wide therapeutic window and species-compatible engineering (e.g., chimerization) further enhance translational feasibility, while rationally designed antibody cocktails can overcome the narrow strain coverage of individual nAbs.

## CLINICAL TRANSLATIONAL POTENTIAL OF nAb(s)

**Fig 5:**
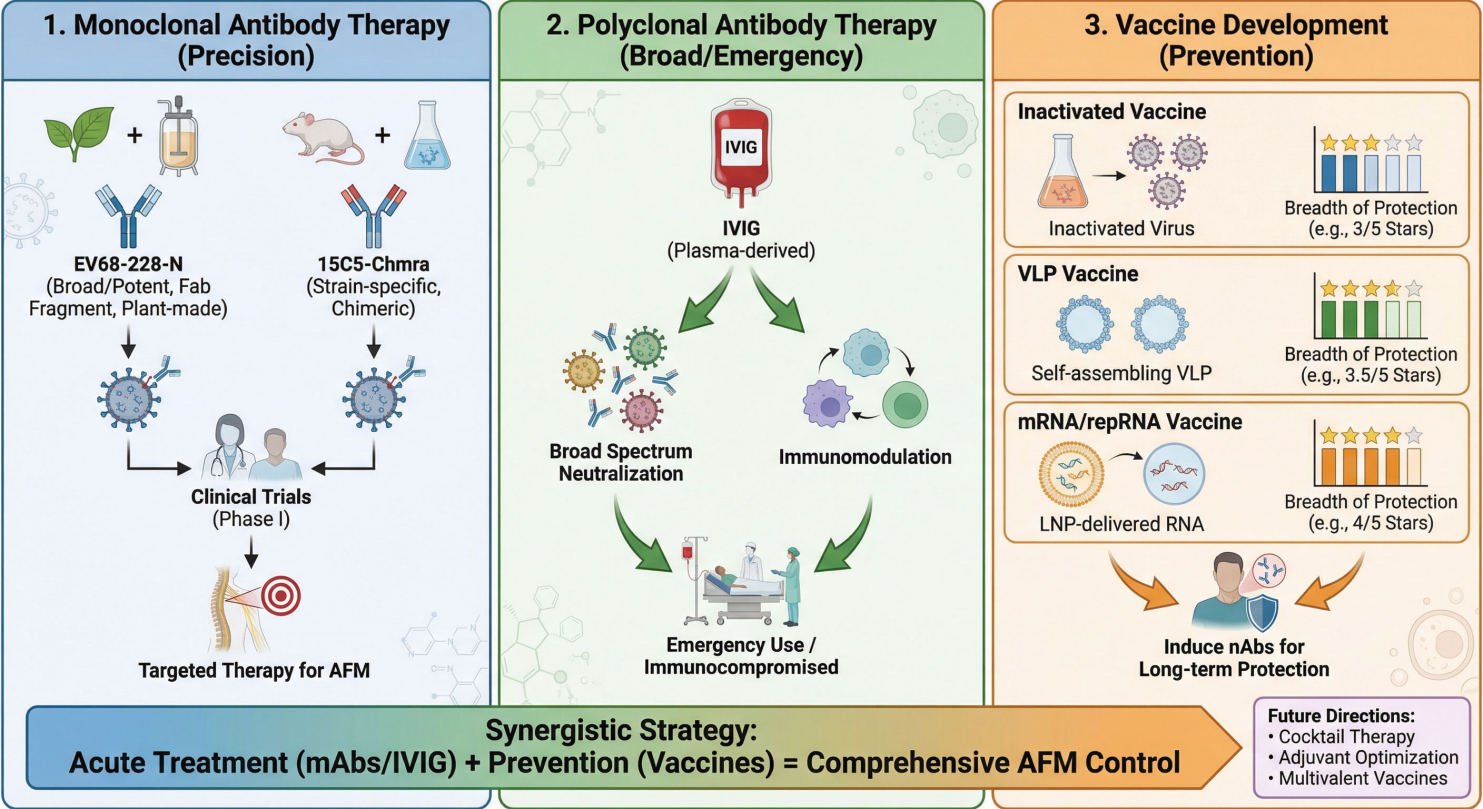
Synergistic strategy of severe EV-D68 treatment.

The promising preclinical efficacy of nAbs against EV-D68 has established a strong rationale for their development as both therapeutic agents and potential vaccine immunogens (Fig. 5). Current translational strategies are primarily focused on two categories of antibody-based products: mAbs and polyclonal antibody preparations. Research on the utilization strategies of natural polyclonal antibodies, namely intravenous immunoglobulin (IVIG), is also an important direction (Table 2).

**TABLE 2:** Comparison of polyclonal antibodies, IVIG, and mAbs against EV-D68

Comparative parameter	Polyclonal antibodies/IVIG	mAbs (e.g., 15C5-Chmra)	Complementarity and rationale for combination
Source and composition	Pooled human plasma; multi-epitope targeting	Recombinant (e.g., chimeric); single epitope, homogeneous	Balance breadth (IVIG) and specificity (mAbs)
Neutralization breadth	Broad (clades A–D); resilient to antigenic drift	High potency for matched strains; susceptible to epitope mutation	IVIG: first-line variant-resistant therapy; mAbs: target prevalent strains
Neutralization potency	Variable (IC_50_: 0.22–85.01 μg/mL)	High & consistent (IC_50_: ~0.01 µg/mL for susceptible strains)	mAbs: rapid high-concentration neutralization; IVIG: sustained broad support
Therapeutic window	Optimal early; moderates progression	Effective post-paralysis onset (≤24 h); prevents spread	IVIG: early/empiric use; mAbs: severe/progressive disease
Mechanism of action	Dual (antiviral neutralization + immunomodulation)	Single (direct viral neutralization via capsid binding)	Synergize to target infection + inflammation
Clinical accessibility	Commercially available; no strain typing needed patients.	Investigational; requires epitope-strain matching	IVIG: rapid emergency use; mAbs: precision severe-case therapy
Key limitations	Donor-dependent; batch variability	Viral escape risk; mismatched strain inactivity	Combined strategy mitigates individual weaknesses

### Translation potential of mAbs

Among neutralizing mAbs being evaluated for EV-D68-associated AFM, both EV68-228-N and 15C5-Chmra have demonstrated post-symptomatic efficacy in mouse models; however, they differ markedly in potency, breadth, and stability (21, 22).

EV68-228-N, a plant-expressed antibody targeting a conserved canyon epitope near the 5-fold axis, exhibits broad and potent neutralization (22). It effectively inhibited circulating strains from 2014 to 2022 (subclades B1, B2, and D), with IC₅₀ values consistently below 330 ng/mL and no observed resistance. In symptomatic neonatal Swiss Webster mice, administration at paralysis onset (score ≥1.5) halted disease progression within 0.76  ±  0.21 days, prevented contralateral and forelimb involvement, and significantly reduced spinal cord and muscle viral loads to the detection limit (158 TCID₅₀/mL). The antibody also preserved lumbar motor neuron counts (16.4  ±  2.3 vs. control) and showed dose-responsive efficacy from 1 to 10 mg/kg. EV68-228-N has advanced to a Phase I clinical trial (NCT06444048), highlighting its potential translational profile.

In contrast, the mouse-human chimeric antibody 15C5-Chmra, which binds a 3-fold plateau epitope, showed more restricted activity (21). While it reduced paralysis severity, improved survival, and lowered viral loads in mice infected with 2014 and 2016 strains when given after paralysis onset (score ≥2), its potency varied substantially by strain: the IC₅₀ for a 2014 isolate was ~0.01  mg/mL, but neutralization of a 2016 strain required >100-fold higher concentrations, and a 2018 isolate was not neutralized even at 1,000 mg/mL. Disease progression was slowed but not reversed, and weight recovery was inconsistent. Overall, 15C5-Chmra demonstrated a narrower breadth and lower *in vivo* efficacy compared to EV68-228-N.

The core findings of the two studies collectively provide critical experimental evidence for targeted therapy of AFM, elucidating the pivotal mechanism by which monoclonal antibodies neutralize the virus and inhibit its spread to healthy motor neurons. Both studies support the clinical principle that “AFM patients with suspected EV-D68 infection should receive treatment as early as possible.”

### Translation potential of polyclonal antibodies and IVIG application

The highly specific EV-68 polyclonal antibody is theoretically the best choice, although no animal experiments have been conducted yet. IVIG, a polyclonal preparation derived from pooled human plasma, represents a clinically available therapeutic option for EV-D68-associated illness. Its broad content of pathogen-specific antibodies offers combined antiviral and immunomodulatory functions. Studies across multiple countries have confirmed the broad-spectrum neutralizing activity of commercial IVIG products against diverse EV-D68 strains (clades A–C), with IC₅₀ values ranging from 0.22 to 85.01  μg/mL and neutralization titers between log₂ 9.5 and log₂ 17.5 (30, 31). Neutralization potency varies among strains within the same clade, correlating with sequence diversity around key antigenic sites (30, 31). *In vivo*, IVIG reduces viral loads in the spinal cord and muscle, improves motor function, and achieves 100% survival in murine models, especially when administered early (32).

Clinically, IVIG is widely used in AFM management, with 73% of patients receiving it alongside glucocorticoids and plasma exchange, although standardized regimens are lacking (33). Its therapeutic relevance is supported by the temporal link between AFM and EV-D68 outbreaks, plus direct viral detection in patients (33). IVIG offers advantages: rapid deployment without strain typing, polyclonal composition mitigating escape risks, and dual antiviral-immunomodulatory effects benefiting immunocompromised individuals (30, 31, 33).

However, critical challenges persist. First, prominent batch-to-batch variability—derived from pooled donor plasma, EV-D68-neutralizing antibody concentration/specificity—varies drastically across batches. IVIG’s IC50 against EV-D68(2076 strain) ranges from 8.05 to 85.01 μg/mL, with 10-fold titer differences, stemming from donor demographics, plasma collection timing, and manufacturing processes (31). This hinders efficacy prediction for AFM patients, especially against emerging variants. Second, non-standardized dosing/duration: empirical use involves 2 g/kg single doses or 0.4 g/kg/day for 5 days, with variable boosters and administration timing (33). Third, evidence relies on retrospective analyses and small case series, with no large prospective trials confirming efficacy (33). Consequently, the CDC’s guidance neither recommends nor discourages IVIG for AFM due to insufficient evidence. Furthermore, patient selection and variant-related potency reduction are still unclear (30). Notably, IVIG neutralizes EV-D68 and heterologous enteroviruses via conserved epitopes, aiding AFM of uncertain etiology (34). Future efforts should focus on prospective trials, manufacturing standardization, dosing guidelines, and refined animal models.

## OTHER ANTIVIRAL THERAPIES

Beyond neutralizing antibody-based approaches, recent advances have led to the exploration of multiple complementary therapeutic directions—including small-molecule inhibitors targeting viral replication and host factors, pyroptosis pathway modulators, and drug repurposing—together forming a multi-faceted therapeutic landscape for combating EV-D68 infection.

### Small-molecule inhibitors: targeting viral and host factors

Small-molecule inhibitors have emerged as a versatile therapeutic direction, with research progressing from viral protein targeting to host factor modulation, each offering distinct mechanisms to counter EV-D68.

The 3C protease of EV-D68 serves as a key target for inhibitor development. Computational biology approaches have identified natural inhibitors such as Withaferin-A and baicalin, which exhibit stable binding to the target with low toxicity and favorable drug development potential, outperforming some synthetic inhibitors (35). However, further *in vitro* and *in vivo* activity validation is required. In contrast, the capsid inhibitor pleconaril holds potential value for severe EV-D68 infections but is limited by drug interactions and viral resistance (36).

Host factors have also emerged as novel targets for antiviral development due to their low mutation rates and minimal drug resistance risks. Phosphatidylinositol 4-kinase IIIβ (PI4KIIIβ) is critical for EV-D68 replication, and novel inhibitors that specifically bind this target exhibit broad-spectrum antiviral activity with extremely low toxicity (37). Additionally, oxysterol-binding protein (OSBP) modulators—such as the FDA-approved antifungal drug itraconazole—can block relevant substance exchange to inhibit viral replication, demonstrating well-established safety profiles and rapid translational potential (38).

### Pyroptosis pathway modulators

A novel therapeutic direction has emerged from the discovery that EV-D68 selectively regulates pyroptosis—a pro-inflammatory programmed cell death pathway—to promote viral replication and pathogenesis (39). Specifically, EV-D68’s 3C protease cleaves gasdermin D (GSDMD) at Q193 to inactivate antiviral pyroptotic responses, while its 2A protease induces gasdermin E (GSDME)-mediated pyroptosis in a cell-type-dependent manner (prominent in GSDME-high HeLa cells but not HEK293T cells).

### Drug repurposing

Drug repurposing accelerates therapeutic development by leveraging clinically approved agents with established safety profiles, offering a rapid path to potential EV-D68 treatments. For instance, the antidepressant fluoxetine inhibits EV-D68 replication by targeting the viral 2C protein, and its higher cerebrospinal fluid concentrations make it particularly suitable for neurological infections associated with AFM (40). However, a clinical trial showed fluoxetine was well-tolerated but provided no neurologic benefit in EV-D68-associated AFM, lacking efficacy despite promising *in vitro* and CNS properties (41). The antimalarial mefloquine and diuretic amiloride also show *in vitro* anti-EV activity, although mefloquine requires formulation optimization to enhance *in vivo* efficacy (40). Additionally, the glutathione synthesis inhibitor buthionine sulfoximine (BSO) interferes with viral capsid assembly and has demonstrated safety in cancer clinical trials, supporting its potential for antiviral application (38).

Despite these advances, EV-D68 non-antibody antiviral therapies face key challenges: viral antigenic drift, limitations in blood-brain barrier penetration, a lack of standardized preclinical models, and unclear immunological correlates of protection for AFM. Future priorities include optimizing inhibitor pharmacokinetics and CNS delivery, developing agents focused on conserved targets, exploring combination therapies, and conducting large-scale pediatric trials.

Drug repurposing accelerates therapeutic development by leveraging clinically approved agents with established safety profiles, offering a rapid path to potential EV-D68 treatments. For instance, the antidepressant fluoxetine inhibits EV-D68 replication by targeting the viral 2C protein, and its ability to concentrate at approximately 20-fold higher levels in the central nervous system (CNS) relative to serum makes it particularly suitable for neurological infections associated with acute flaccid myelitis (AFM) (40).

## VACCINE DEVELOPMENT

EV-D68 vaccine development currently centers on three principal platforms: inactivated vaccines, VLP vaccines, and nucleic acid vaccines. All aim to elicit potent neutralizing antibodies, yet each presents distinct advantages and challenges on the path to clinical translation (Table 3).

**TABLE 3:** Comparison table of core data of different vaccine platforms for EV-D68

Vaccine platform	Expression/production system	Animal model	Cross-neutralization spectrum	Protective efficacy *in vivo*	Limitations/future directions
Mucosal bivalent inactivated vaccine	Vero cell culture, formalin inactivation, ganoderma polysaccharide (PS-G) adjuvant	ICR neonatal and adult mice	EV-D68 (B1/B3/A2); EV-A71 (multiple subclades)	100% protection in neonates; blocks CNS invasion; mucosal sIgA persists 16–19 weeks	Duration of mucosal immunity; pediatric clinical validation needed
VLP vaccine (Pichia pastoris)	pastoris co-expression of P1 + 3 CD	ICR neonatal and adult mice	EV-D68 (A/B1/Fermon)	100% survival in neonates (passive and maternal); blocks muscle/spinal cord replication	Limited protection vs. B3/D clades; immunization route optimization
VLP vaccine (Baculovirus-Insect Cell)	Baculovirus/Sf9 co-expression of P1 + 3 CD	ICR neonatal and adult mice	EV-D68 (A/B1/Fermon)	100% neonatal survival; clears respiratory virus	Complex production; immunogenicity in NHPs required
Vero-adapted inactivated vaccine	Vero cell adaptation, formalin inactivation ± alum adjuvant	BALB/c and ICR neonatal mice	EV-D68 (A/B1/Fermon)	100% protection; reduces tissue viral load by 3–6 log	Seven adaptive mutations in strain; human dosing studies needed
Cotton rat inactivated vaccine	HeLa cell culture, live/UV-inactivated virus	Sigmodon hispidus (cotton rat)	EV-D68 (A1/B1/ATCC prototype)	Blocks respiratory replication; reduces lung inflammation; nAbs > 9 weeks	Does not induce nAbs; potential immune enhancement risk
Rhesus-derived inactivated vaccine	Vero cell culture, formalin inactivation, alum adjuvant	Rhesus macaque, C57BL/6 neonatal mice	EV-D68 (KM/Fermon)	100% neonatal survival via maternal antibodies; prevents paralysis after IC challenge	Macaque cohort size; pediatric immunogenicity validation

**Fig 6:**
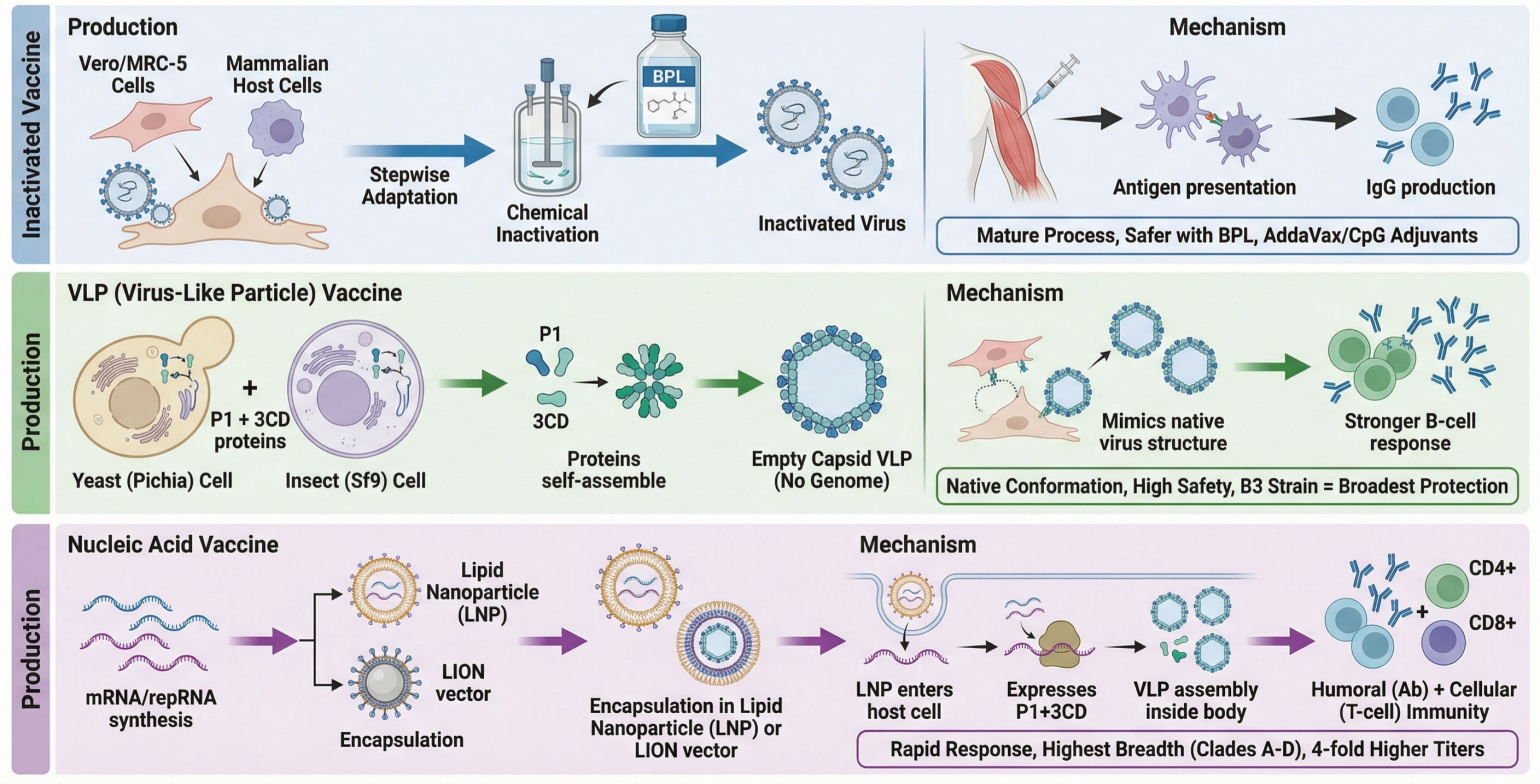
EV-D68 vaccine platforms: production and mechanism.

Current EV-D68 vaccine strategies are built upon three principal technological platforms: inactivated vaccines, virus-like particle (VLP) vaccines, and nucleic acid vaccines. Each platform aims to elicit potent nAb responses and possesses distinct advantages and challenges as they advance toward clinical application (Fig. 6).

### Inactivated vaccines

Inactivated vaccines, with their mature production processes and well-established safety profiles, have become the predominant approach in the early-stage development of EV-D68 vaccines. The core technological pathway involves culturing the virus in a compliant cell matrix, followed by inactivation to retain neutralizing epitopes, thereby inducing the production of protective antibodies in the host (42–45).

In terms of cell matrix optimization, the traditionally relied-upon tumor-derived RD cells have potential safety risks. The research team successfully developed EV-D68 virus strains adapted to MRC-5 and Vero cells (the World Health Organization-approved vaccine production cell lines) through a “stepwise adaptation” strategy, with the highest virus titer reaching 10^8.3^ TCID_50_/mL. Moreover, some adapted strains (such as the MO strain) retained the high immunogenicity of the parent strain (42). Screening of inactivation reagents showed that β-propiolactone (BPL) caused the least damage to the viral antigen structure, and its quality control was easier to standardize. It was superior to formalin and hydrogen peroxide, thus becoming the optimal inactivation choice (44). For adjuvantation, CpG ODN and AddaVax increased nAb titers by 2-fold to 3-fold, whereas traditional aluminum hydroxide showed no significant enhancement, indicating that adjuvant selection must be matched to the properties of the inactivated virus (43, 45).

In terms of immune efficacy, the nAb titers induced by inactivated vaccines ranged from 1:64 to 1:11,585, demonstrating cross-coverage of subclades A and B (43–45). The protective effect of intramuscular injection was significantly superior to mucosal administration, effectively blocking viral respiratory replication and neural invasion (43, 45, 46). Maternal antibody transfer experiments confirmed that the survival rate of offspring lactating mice from immunized sows reached 100%, effectively preventing limb paralysis (44, 46). However, such vaccines still have limitations: heterologous strain protection is limited, and some strains may disrupt key neutralizing epitopes during adaptation. Additionally, the inactivation process may affect antigen conformation integrity (36, 42, 43).

### VLP vaccines

VLP vaccines, which express recombinant viral structural proteins and self-assemble into naturally conformational particles, exhibit both safety and high immunogenicity, making them a key direction in EV-D68 vaccine development. The primary expression systems include Pichia pastoris and baculovirus-insect cell systems (44, 47).

The Pichia pastoris expression system achieves precise cleavage and self-assembly of VP0, VP1, and VP3 proteins by co-expressing the P1 capsid precursor protein and 3CD protease, generating VLPs with a diameter of approximately 30 nm. This system has low fermentation costs and is easy for large-scale production. The geometric mean titer (GMT) of the induced neutralizing antibodies reaches log₂1149, which can cross-neutralize subclade A and the prototype strain. Both passive immunity and maternal transmission can achieve 100% protection of suckling mice (44). The baculovirus-insect cell system exhibits better antigen assembly efficiency, with VLP structures highly consistent with natural viruses. The GMT of neutralizing antibodies is as high as log_2_4598, and the cross-neutralization spectrum covers subclade A, subclade B1, and the prototype strain. The virus clearance efficiency is significantly better than that of some inactivated vaccines (47).

Strain selection is critical for the efficacy of VLP vaccines. The B3 strain VLP exhibits 1–8 times higher protective potency against subclades A2 and B1 compared to the B1 strain, better aligning with current epidemiological needs (48). When combined with the Adjuplex adjuvant, it induces a balanced Th1/Th2 immune response, mitigating the risk of respiratory inflammation associated with unbalanced Th2 responses (48). However, VLP vaccines face challenges, such as higher production complexity than inactivated vaccines and insufficient cross-protection against subclades B3/D, necessitating further optimization of large-scale production techniques and antigen epitope presentation (44, 47, 48).

### Nucleic acid vaccines

Nucleic acid vaccines (mRNA/repRNA vaccines), as an emerging technological approach in recent years, have become an innovative breakthrough in EV-D68 vaccine development due to their advantages of rapid preparation and high immunogenicity. The core mechanism involves vector-delivered viral antigen-coding sequences, which are expressed *in vivo* to induce immune responses (49, 50).

The mRNA vaccine uses a dual mRNA-LNP system to express P1 precursor and 3CD protease, assembling VLPs with exposed neutralizing epitopes (44). In mice, it induced nAb titer of 1,024 (4× that of inactivated vaccines), covering subclades A–D, with higher mucosal antibodies to block respiratory infection and neuroinvasion (49). The repRNA vaccine employs cationic nanocarrier LION to avoid LNP-induced systemic inflammation. In rhesus monkeys, it elicited cross-neutralizing antibodies with 11.3× lower serum IFN-α2, improving safety (50, 51). The single-stranded self-replicating RNA vaccine concatenates P1/3CD via IRES and combines with LION, inducing nAb titer up to 1:2,124 in rhesus monkeys with superior cross-neutralization for six subclades (51, 52). It adapts rapidly to viral mutations and enables multivalent development but requires verification of pediatric immunogenicity and long-term safety (51).

Inactivated, VLP, and nucleic acid vaccines for EV-D68 complement each other with pros and cons. Inactivated vaccines suit rapid industrialization but have narrow cross-protection. VLP vaccines balance safety and broad-spectrum efficacy yet face high production complexity. Nucleic acid vaccines offer optimal broad-spectrum coverage and quick response but lack sufficient clinical data. VLP and nucleic acid vaccines induce higher neutralizing antibody titers and better cross-neutralization than inactivated ones, with nucleic acid vaccines additionally stimulating cellular immunity. Future R&D should focus on multivalent vaccine development, adjuvant/delivery system optimization, and pediatric adaptation. Combining vaccines with antiviral drugs is promising (53). EV-D68 vaccines are expected to enter clinical use soon, while currently, none of the EV-D68 vaccine candidates have entered clinical trials or obtained human clinical data.

## CHALLENGES AND FUTURE DIRECTIONS

Despite considerable advances, the development of EV-D68 countermeasures faces several persistent challenges. First, ongoing viral evolution and antigenic drift threaten the durability of both nAbs and vaccines. Sustained surveillance of circulating genotypes and epitope landscapes is essential to guide updates to antibody cocktails and vaccine antigens. Second, the BBB restricts antibody access to the CNS, the principal site of viral replication in AFM. Enhancing CNS delivery—for instance, through BBB-penetrating peptide conjugates—represents a priority for treating neurological complications. Third, the immunological correlates of protection against AFM remain poorly defined. While nAb titers correlate with protection from respiratory infection, their role in preventing neuroinvasion is less clear, warranting investigation into complementary roles for T-cell responses and mucosal immunity. Finally, the high cost of monoclonal antibody production and limited global access to IVIG underscore the need for affordable interventions, such as low-cost VLP-based vaccines or orally administered platforms.

Future efforts should prioritize (i) identifying conserved, immunogenic epitopes to design broad-spectrum vaccines, (ii) engineering antibody or delivery platforms that enhance CNS penetration, and (iii) developing antibody cocktails targeting non-overlapping epitopes to minimize viral escape. Large-scale clinical trials, particularly in pediatric populations, will be essential to validate the safety and efficacy of mAbs and next-generation vaccines against EV-D68.
